# 1-{(*E*)-[3-(1*H*-Imidazol-1-yl)-1-(4-meth­oxy­phen­yl)propyl­idene]amino}-3-(2-methyl­phen­yl)urea

**DOI:** 10.1107/S1600536812021903

**Published:** 2012-05-19

**Authors:** Mohamed I. Attia, Mohamed N. Aboul-Enein, Nasser R. El-Brollosy, Seik Weng Ng, Edward R. T. Tiekink

**Affiliations:** a Department of Pharmaceutical Chemistry, College of Pharmacy, King Saud University, Riyadh 11451, Saudi Arabia; b Medicinal and Pharmaceutical Chemistry Department, Pharmaceutical and Drug Industries Research Division, National Research Centre, 12622, Dokki, Giza, Egypt; c Department of Chemistry, University of Malaya, 50603 Kuala Lumpur, Malaysia; d Chemistry Department, Faculty of Science, King Abdulaziz University, PO Box 80203 Jeddah, Saudi Arabia

## Abstract

In the title compound, C_21_H_23_N_5_O_2_, the conformation about the imine bond [1.287 (3) Å] is *E*. Overall, the mol­ecule has a disk shape, the dihedral angles between the imidazole ring and the meth­oxy­phenyl and methyl­phenyl rings being 49.42 (13) and 42.62 (13)°, respectively; the dihedral angle between the benzene rings is 20.11 (11)°. In the urea moiety, the N—H atoms are *anti* to each other and one of these forms an intra­molecular N—H⋯N hydrogen bond. In the crystal, centrosymmetric dimers are formed *via* N—H⋯N(imidazole) hydrogen bonds, which are connected into a three-dimensional architecture by C—H⋯O(carbon­yl) and (methyl­ene)C—H⋯π inter­actions. The crystal studied was a non-merohedral twin with a minor component of 48.3 (1)%.

## Related literature

For background to the prevalence of epilepsy and epilepsy drugs, see: Sander & Shorvon (1987 ▶); Saxena & Saxena (1995 ▶); Edafiogho & Scott (1996 ▶). For the use of aryl semicarbazones as anti-convulsants, see: Aboul-Enein *et al.* (2012 ▶); Dimmock *et al.* (1993 ▶, 1995 ▶). For a related structure, see: Attia *et al.* (2012 ▶).
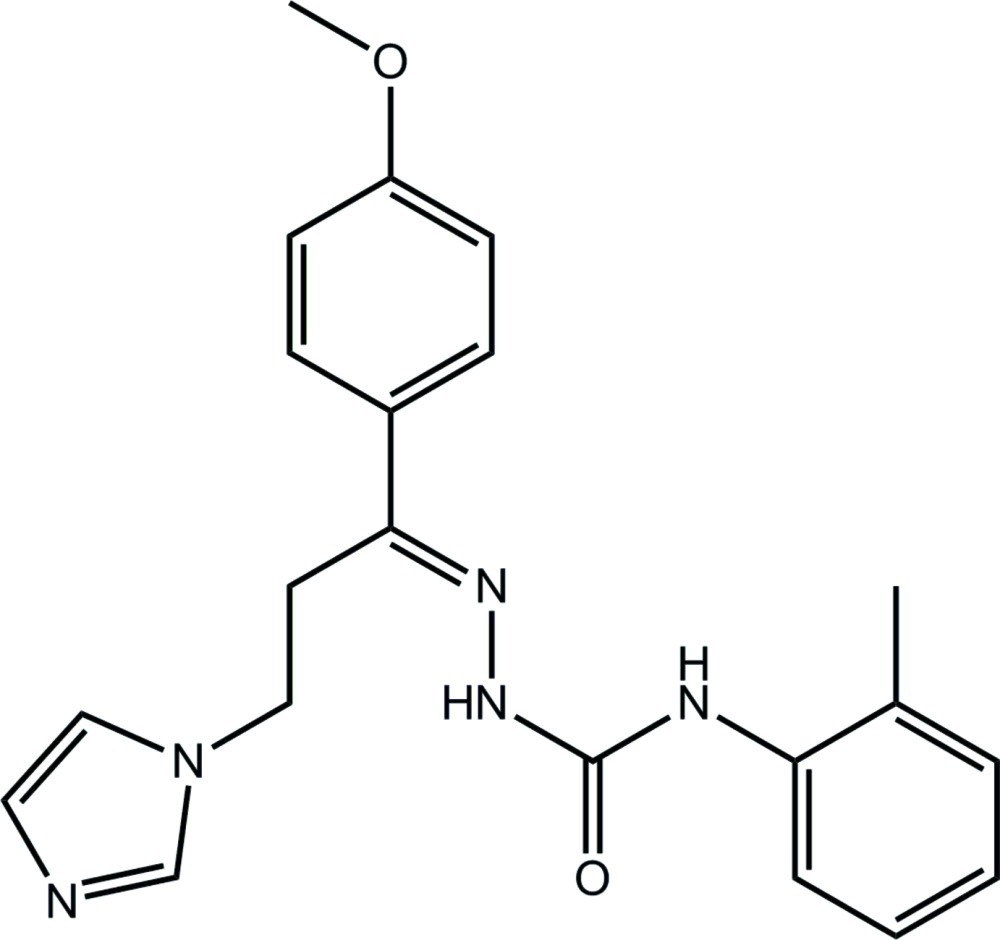


## Experimental

### 

#### Crystal data

C_21_H_23_N_5_O_2_
*M* *_r_* = 377.44
Monoclinic, 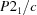

*a* = 10.7798 (12) Å
*b* = 20.7750 (19) Å
*c* = 8.7652 (18) Å
β = 105.318 (15)°
*V* = 1893.2 (5) Å^3^
*Z* = 4
Mo *K*α radiation
μ = 0.09 mm^−1^
*T* = 100 K
0.35 × 0.15 × 0.03 mm

#### Data collection

Agilent SuperNova Dual diffractometer with an Atlas detector
Absorption correction: multi-scan (*CrysAlis PRO*; Agilent, 2011 ▶) *T*_min_ = 0.692, *T*_max_ = 1.000
15110 measured reflections
7494 independent reflections
4657 reflections with *I* > 2σ(*I*)
*R*_int_ = 0.080

#### Refinement

*R*[*F* ^2^ > 2σ(*F* ^2^)] = 0.064
*wR*(*F* ^2^) = 0.216
*S* = 0.98
7494 reflections
263 parameters
H atoms treated by a mixture of independent and constrained refinement
Δρ_max_ = 0.31 e Å^−3^
Δρ_min_ = −0.32 e Å^−3^

### 

Data collection: *CrysAlis PRO* (Agilent, 2011 ▶); cell refinement: *CrysAlis PRO*; data reduction: *CrysAlis PRO*; program(s) used to solve structure: *SHELXS97* (Sheldrick, 2008 ▶); program(s) used to refine structure: *SHELXL97* (Sheldrick, 2008 ▶); molecular graphics: *ORTEP-3* (Farrugia, 1997 ▶) and *DIAMOND* (Brandenburg, 2006 ▶); software used to prepare material for publication: *publCIF* (Westrip, 2010 ▶).

## Supplementary Material

Crystal structure: contains datablock(s) global, I. DOI: 10.1107/S1600536812021903/gg2079sup1.cif

Structure factors: contains datablock(s) I. DOI: 10.1107/S1600536812021903/gg2079Isup2.hkl

Supplementary material file. DOI: 10.1107/S1600536812021903/gg2079Isup3.cml

Additional supplementary materials:  crystallographic information; 3D view; checkCIF report

## Figures and Tables

**Table 1:** Hydrogen-bond geometry (Å, °) *Cg*2 and *Cg*3 are the centroids of the C1–C6 and C10–C15 benzene rings, respectively.

*D*—H⋯*A*	*D*—H	H⋯*A*	*D*⋯*A*	*D*—H⋯*A*
N1—H1*n*⋯N3	0.87 (3)	2.04 (2)	2.568 (3)	118 (2)
N2—H2*n*⋯N5^i^	0.87 (3)	2.17 (3)	3.029 (3)	171 (2)
C16—H16*B*⋯O1^ii^	0.98	2.44	3.398 (3)	165
C20—H20⋯O1^i^	0.95	2.51	3.226 (3)	133
C17—H17*A*⋯*Cg*2^iii^	0.99	2.80	3.391 (3)	119
C18—H18*B*⋯*Cg*3^iv^	0.99	2.78	3.569 (2)	137

Symmetry codes: (i) 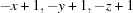; (ii) 
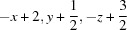
; (iii) 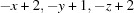; (iv) 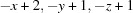.

**Fig. 1.**
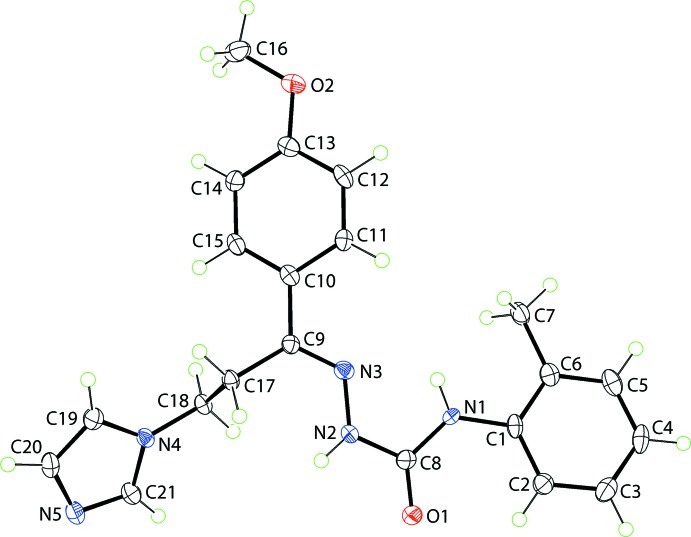
The molecular structure of (I) showing the atom-labelling scheme and displacement ellipsoids at the 50% probability level.

**Fig. 2.**
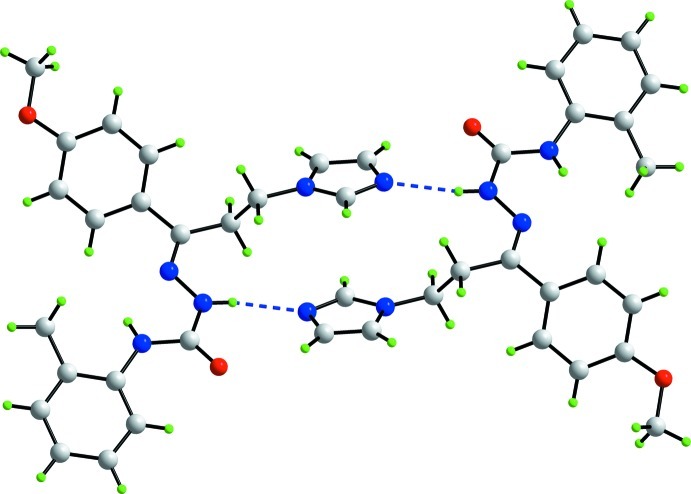
A view of the supramolecular dimer in (I) mediated by N—H···N hydrogen bonding, shown as blue dashed lines.

**Fig. 3.**
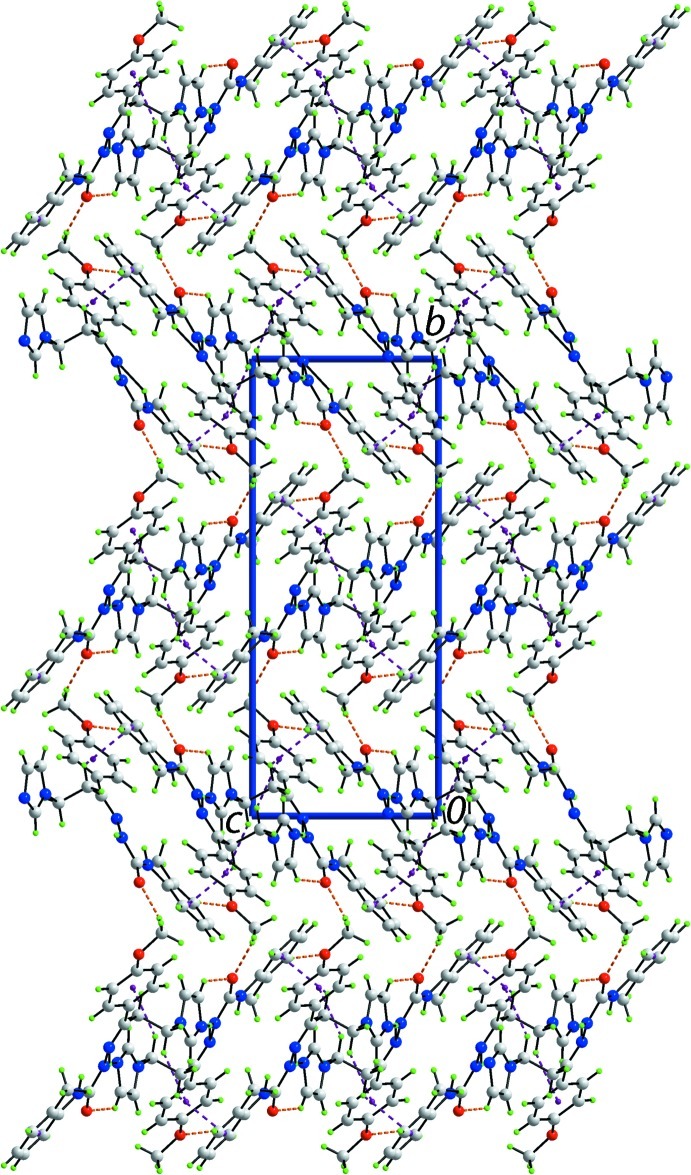
A view in projection down the a axis of the unit-cell contents for (I). The N—H···N, C—H···O and C—H···π interactions are shown as blue, orange and purple dashed lines, respectively.

## supplementary crystallographic information

### Comment

Epilepsy is one of the most widespread pathologies of the human brain, affecting approximately 1% of world population (Sander & Shorvon, 1987). Current anti-epileptic drugs suffer from a number of disadvantages including the fact that approximately one quarter of epileptic patients have seizures that are resistant to the available medical therapy (Saxena & Saxena, 1995). Additionally, many clinically used anti-epileptic drugs cause significant side-effects which may limit their usefulness (Edafiogho & Scott, 1996). Accordingly, the evolution of novel anti-convulsants is a continuing challenge. An evaluation of the literature revealed that aryl semicarbazones were found to exhibit significant anti-convulsant activities (Aboul-Enein *et al.*, 2012; Dimmock *et al.*, 1995; Dimmock *et al.*, 1993). The novel title compound, namely (2*E*)-2-[3-(1*H*-imidazol-1-yl)-1-(4-methoxphenyl)propylidene]-*N*-(2-methylphenyl)hydrazinecarboxamide (I) will be evaluated as anti-convulsant in experimental animal models. Herein, we describe the results of its crystal structure determination.

In (I), Fig. 1, the conformation about the N3═C9 bond [1.287 (3) Å] is *E*. The dihedral angles between the imidazolyl ring and the methoxy- and methyl-benzene rings are 49.42 (13) and 42.62 (13)°, respectively; the dihedral angle between the benzene rings is 20.11 (11)°. Despite these angles of inclination, overall the molecule as a disk which contrasts the flat topology in the non-methoxy species (Attia *et al.*, 2012). The methoxy group is co-planar with the benzene ring to which it is attached as seen in the value of the C16—O2—C13—C12 torsion angle of -173.1 (2)°. Within the urea moiety, the N—H atoms are *anti* to each other and the N1—H forms an intramolecular N—H···N hydrogen bond to define a *S*(5) loop, Table 1.

In the crystal structure, centrosymmetric dimers are formed *via* N—H···N(imidazolyl) hydrogen bonds and 18-membered {···HNNC_3_NCN}_2_ synthons, Fig. 2 and Table 1. These aggregates are connected into a three-dimensional architecture by *C*—*H*···*O*(carbonyl) and (methylene)C—H···π interactions, Fig. 3 and Table 1.

### Experimental

Acetic acid (2 drops) was added to a stirred solution of 3-(1*H*-imidazol-1-yl)-1-(4-methoxyphenyl)propan-1-one (0.23 g, 1 mmol) and *N*-(2-methylphenyl)hydrazinecarboxamide (0.17 g, 1 mmol) in absolute ethanol (10 ml). The reaction mixture was stirred at room temperature for 18 h. The solvent was concentrated under reduced pressure and the precipitated solid was collected by filtration. The collected solid was recrystallized from ethanol to give crystals of the title compound; *Mp*: 363–365 K.

### Refinement

Carbon-bound H-atoms were placed in calculated positions [C—H = 0.95 to 0.99 Å, *U*_iso_(H) = 1.2–1.5*U*_eq_(C)] and were included in the refinement in the riding model approximation. The amino H-atoms were refined freely. The crystal studied was a non-merohedral twin with the minor component being 48.3 (1)%. The (6 9 0) reflection was omitted owing to poor agreement.

### Crystal data

**Table:**

C_21_H_23_N_5_O_2_	*F*(000) = 800
*M**_r_* = 377.44	*D*_x_ = 1.324 Mg m^−^^3^
Monoclinic, *P*2_1_/*c*	Melting point: 364 K
Hall symbol: -P 2ybc	Mo *K*α radiation, λ = 0.71073 Å
*a* = 10.7798 (12) Å	Cell parameters from 1904 reflections
*b* = 20.7750 (19) Å	θ = 2.4–27.5°
*c* = 8.7652 (18) Å	µ = 0.09 mm^−^^1^
β = 105.318 (15)°	*T* = 100 K
*V* = 1893.2 (5) Å^3^	Prism, colourless
*Z* = 4	0.35 × 0.15 × 0.03 mm

### Data collection

**Table:**

Agilent SuperNova Dual diffractometer with an Atlas detector	7494 independent reflections
Radiation source: SuperNova (Mo) X-ray Source	4657 reflections with *I* > 2σ(*I*)
Mirror monochromator	*R*_int_ = 0.080
Detector resolution: 10.4041 pixels mm^-1^	θ_max_ = 27.7°, θ_min_ = 2.6°
ω scan	*h* = −12→14
Absorption correction: multi-scan (*CrysAlis PRO*; Agilent, 2011)	*k* = −27→27
*T*_min_ = 0.692, *T*_max_ = 1.000	*l* = −11→11
15110 measured reflections	

### Refinement

**Table:**

Refinement on *F*^2^	Primary atom site location: structure-invariant direct methods
Least-squares matrix: full	Secondary atom site location: difference Fourier map
*R*[*F*^2^ > 2σ(*F*^2^)] = 0.064	Hydrogen site location: inferred from neighbouring sites
*wR*(*F*^2^) = 0.216	H atoms treated by a mixture of independent and constrained refinement
*S* = 0.98	*w* = 1/[σ^2^(*F*_o_^2^) + (0.1326*P*)^2^] where *P* = (*F*_o_^2^ + 2*F*_c_^2^)/3
7494 reflections	(Δ/σ)_max_ < 0.001
263 parameters	Δρ_max_ = 0.31 e Å^−^^3^
0 restraints	Δρ_min_ = −0.32 e Å^−^^3^

### Special details

**Table:**

Geometry. All e.s.d.'s (except the e.s.d. in the dihedral angle between two l.s. planes) are estimated using the full covariance matrix. The cell e.s.d.'s are taken into account individually in the estimation of e.s.d.'s in distances, angles and torsion angles; correlations between e.s.d.'s in cell parameters are only used when they are defined by crystal symmetry. An approximate (isotropic) treatment of cell e.s.d.'s is used for estimating e.s.d.'s involving l.s. planes.
Refinement. Refinement of *F*^2^ against ALL reflections. The weighted *R*-factor *wR* and goodness of fit *S* are based on *F*^2^, conventional *R*-factors *R* are based on *F*, with *F* set to zero for negative *F*^2^. The threshold expression of *F*^2^ > σ(*F*^2^) is used only for calculating *R*-factors(gt) *etc*. and is not relevant to the choice of reflections for refinement. *R*-factors based on *F*^2^ are statistically about twice as large as those based on *F*, and *R*- factors based on ALL data will be even larger.

### Fractional atomic coordinates and isotropic or equivalent isotropic displacement parameters (Å^2^)

**Table:**

	*x*	*y*	*z*	*U*_iso_*/*U*_eq_	
O1	0.84974 (15)	0.35509 (7)	0.8918 (2)	0.0223 (4)	
O2	1.35476 (15)	0.69761 (8)	0.6108 (2)	0.0274 (4)	
N1	1.05626 (18)	0.39321 (9)	0.9372 (2)	0.0179 (5)	
H1n	1.091 (2)	0.4294 (12)	0.919 (3)	0.024 (7)*	
N2	0.88515 (19)	0.45303 (9)	0.7984 (3)	0.0192 (5)	
H2n	0.803 (3)	0.4582 (11)	0.770 (3)	0.020 (7)*	
N3	0.97616 (17)	0.49398 (9)	0.7705 (3)	0.0181 (4)	
N4	0.61494 (17)	0.54805 (8)	0.3870 (2)	0.0166 (4)	
N5	0.40525 (18)	0.53958 (9)	0.2745 (3)	0.0230 (5)	
C1	1.1287 (2)	0.34837 (10)	1.0450 (3)	0.0173 (5)	
C2	1.0727 (2)	0.30283 (10)	1.1230 (3)	0.0212 (5)	
H2A	0.9817	0.3003	1.1018	0.025*	
C3	1.1496 (2)	0.26135 (11)	1.2310 (3)	0.0261 (6)	
H3	1.1111	0.2306	1.2845	0.031*	
C4	1.2818 (2)	0.26431 (11)	1.2618 (3)	0.0262 (6)	
H4	1.3343	0.2357	1.3360	0.031*	
C5	1.3371 (2)	0.30935 (11)	1.1838 (3)	0.0249 (6)	
H5	1.4281	0.3112	1.2056	0.030*	
C6	1.2633 (2)	0.35195 (11)	1.0743 (3)	0.0199 (5)	
C7	1.3245 (2)	0.40109 (12)	0.9928 (3)	0.0263 (6)	
H7A	1.2901	0.3967	0.8780	0.040*	
H7B	1.4178	0.3944	1.0214	0.040*	
H7C	1.3055	0.4443	1.0255	0.040*	
C8	0.9257 (2)	0.39659 (10)	0.8786 (3)	0.0172 (5)	
C9	0.9418 (2)	0.54534 (9)	0.6881 (3)	0.0156 (5)	
C10	1.0481 (2)	0.58629 (10)	0.6644 (3)	0.0170 (5)	
C11	1.1687 (2)	0.58495 (10)	0.7738 (3)	0.0171 (5)	
H11	1.1824	0.5577	0.8638	0.020*	
C12	1.2674 (2)	0.62245 (11)	0.7526 (3)	0.0202 (5)	
H12	1.3486	0.6212	0.8285	0.024*	
C13	1.2499 (2)	0.66239 (10)	0.6209 (3)	0.0191 (5)	
C14	1.1307 (2)	0.66451 (10)	0.5107 (3)	0.0206 (5)	
H14	1.1175	0.6912	0.4197	0.025*	
C15	1.0311 (2)	0.62687 (10)	0.5357 (3)	0.0201 (5)	
H15	0.9489	0.6292	0.4620	0.024*	
C16	1.3358 (2)	0.74429 (11)	0.4871 (3)	0.0291 (6)	
H16A	1.4176	0.7657	0.4912	0.044*	
H16B	1.2728	0.7763	0.5006	0.044*	
H16C	1.3040	0.7229	0.3845	0.044*	
C17	0.8032 (2)	0.56417 (10)	0.6144 (3)	0.0164 (5)	
H17A	0.7981	0.6112	0.5958	0.020*	
H17B	0.7510	0.5537	0.6886	0.020*	
C18	0.7482 (2)	0.52920 (10)	0.4584 (3)	0.0174 (5)	
H18A	0.8008	0.5393	0.3845	0.021*	
H18B	0.7523	0.4822	0.4772	0.021*	
C19	0.5669 (2)	0.60937 (11)	0.3609 (3)	0.0226 (6)	
H19	0.6141	0.6483	0.3862	0.027*	
C20	0.4397 (2)	0.60370 (11)	0.2921 (3)	0.0235 (6)	
H20	0.3821	0.6388	0.2604	0.028*	
C21	0.5137 (2)	0.50860 (11)	0.3321 (3)	0.0212 (6)	
H21	0.5201	0.4630	0.3348	0.025*	

### Atomic displacement parameters (Å^2^)

**Table:**

	*U*^11^	*U*^22^	*U*^33^	*U*^12^	*U*^13^	*U*^23^
O1	0.0201 (8)	0.0256 (8)	0.0198 (10)	−0.0039 (7)	0.0030 (7)	0.0045 (7)
O2	0.0192 (9)	0.0309 (9)	0.0319 (12)	−0.0056 (7)	0.0066 (8)	0.0031 (8)
N1	0.0172 (10)	0.0152 (9)	0.0202 (12)	−0.0024 (8)	0.0031 (9)	0.0063 (8)
N2	0.0131 (10)	0.0224 (10)	0.0207 (12)	−0.0005 (8)	0.0019 (9)	0.0047 (9)
N3	0.0165 (10)	0.0220 (10)	0.0162 (11)	−0.0015 (8)	0.0051 (8)	−0.0004 (8)
N4	0.0145 (9)	0.0216 (9)	0.0127 (11)	−0.0001 (7)	0.0020 (8)	0.0002 (8)
N5	0.0172 (10)	0.0275 (11)	0.0224 (12)	0.0039 (8)	0.0019 (9)	0.0017 (9)
C1	0.0215 (12)	0.0173 (11)	0.0106 (12)	0.0054 (9)	0.0000 (10)	−0.0029 (9)
C2	0.0232 (13)	0.0213 (12)	0.0179 (14)	−0.0024 (9)	0.0030 (11)	−0.0015 (10)
C3	0.0367 (15)	0.0209 (12)	0.0192 (15)	−0.0027 (10)	0.0050 (12)	0.0019 (10)
C4	0.0346 (14)	0.0200 (12)	0.0194 (15)	0.0101 (10)	−0.0012 (12)	0.0027 (10)
C5	0.0213 (13)	0.0306 (13)	0.0198 (14)	0.0063 (10)	0.0003 (11)	−0.0065 (11)
C6	0.0221 (12)	0.0229 (11)	0.0139 (13)	0.0017 (9)	0.0032 (10)	−0.0041 (10)
C7	0.0155 (12)	0.0370 (14)	0.0252 (15)	0.0038 (10)	0.0031 (11)	0.0067 (12)
C8	0.0206 (12)	0.0212 (11)	0.0104 (12)	−0.0026 (9)	0.0051 (10)	−0.0021 (10)
C9	0.0168 (11)	0.0154 (10)	0.0136 (13)	−0.0004 (9)	0.0018 (9)	−0.0018 (9)
C10	0.0154 (11)	0.0184 (11)	0.0164 (13)	0.0018 (9)	0.0027 (10)	−0.0051 (10)
C11	0.0203 (12)	0.0164 (10)	0.0137 (13)	0.0026 (9)	0.0029 (10)	−0.0013 (9)
C12	0.0131 (11)	0.0256 (12)	0.0198 (14)	0.0044 (9)	0.0009 (10)	−0.0051 (10)
C13	0.0160 (12)	0.0190 (11)	0.0236 (14)	0.0009 (9)	0.0073 (10)	−0.0054 (10)
C14	0.0219 (12)	0.0206 (11)	0.0182 (14)	−0.0002 (9)	0.0036 (10)	0.0028 (10)
C15	0.0148 (11)	0.0216 (11)	0.0213 (14)	0.0028 (9)	0.0000 (10)	−0.0015 (10)
C16	0.0287 (14)	0.0244 (12)	0.0383 (19)	−0.0028 (11)	0.0158 (13)	0.0034 (12)
C17	0.0138 (11)	0.0195 (11)	0.0161 (13)	0.0040 (8)	0.0043 (10)	0.0007 (10)
C18	0.0142 (11)	0.0203 (11)	0.0171 (13)	0.0028 (9)	0.0031 (10)	−0.0008 (10)
C19	0.0221 (12)	0.0188 (11)	0.0263 (15)	0.0020 (9)	0.0050 (11)	0.0015 (11)
C20	0.0212 (13)	0.0247 (12)	0.0233 (15)	0.0077 (10)	0.0034 (11)	0.0034 (11)
C21	0.0182 (12)	0.0247 (12)	0.0189 (15)	0.0008 (9)	0.0017 (11)	−0.0016 (11)

### Geometric parameters (Å, º)

**Table:**

O1—C8	1.216 (3)	C7—H7B	0.9800
O2—C13	1.369 (3)	C7—H7C	0.9800
O2—C16	1.429 (3)	C9—C10	1.485 (3)
N1—C8	1.366 (3)	C9—C17	1.514 (3)
N1—C1	1.408 (3)	C10—C15	1.381 (3)
N1—H1n	0.87 (3)	C10—C11	1.397 (3)
N2—N3	1.369 (3)	C11—C12	1.371 (3)
N2—C8	1.378 (3)	C11—H11	0.9500
N2—H2n	0.87 (3)	C12—C13	1.393 (3)
N3—C9	1.287 (3)	C12—H12	0.9500
N4—C21	1.348 (3)	C13—C14	1.389 (3)
N4—C19	1.371 (3)	C14—C15	1.392 (3)
N4—C18	1.460 (3)	C14—H14	0.9500
N5—C21	1.313 (3)	C15—H15	0.9500
N5—C20	1.381 (3)	C16—H16A	0.9800
C1—C2	1.394 (3)	C16—H16B	0.9800
C1—C6	1.408 (3)	C16—H16C	0.9800
C2—C3	1.383 (3)	C17—C18	1.523 (3)
C2—H2A	0.9500	C17—H17A	0.9900
C3—C4	1.380 (4)	C17—H17B	0.9900
C3—H3	0.9500	C18—H18A	0.9900
C4—C5	1.384 (4)	C18—H18B	0.9900
C4—H4	0.9500	C19—C20	1.349 (3)
C5—C6	1.390 (3)	C19—H19	0.9500
C5—H5	0.9500	C20—H20	0.9500
C6—C7	1.495 (3)	C21—H21	0.9500
C7—H7A	0.9800		
			
C13—O2—C16	117.18 (18)	C11—C10—C9	120.3 (2)
C8—N1—C1	128.3 (2)	C12—C11—C10	120.7 (2)
C8—N1—H1n	109.8 (16)	C12—C11—H11	119.6
C1—N1—H1n	120.2 (17)	C10—C11—H11	119.6
N3—N2—C8	118.31 (19)	C11—C12—C13	120.7 (2)
N3—N2—H2n	126.9 (16)	C11—C12—H12	119.6
C8—N2—H2n	114.7 (16)	C13—C12—H12	119.6
C9—N3—N2	120.13 (19)	O2—C13—C14	124.5 (2)
C21—N4—C19	105.71 (18)	O2—C13—C12	116.0 (2)
C21—N4—C18	127.00 (18)	C14—C13—C12	119.6 (2)
C19—N4—C18	127.29 (18)	C13—C14—C15	118.9 (2)
C21—N5—C20	104.13 (19)	C13—C14—H14	120.6
C2—C1—C6	120.4 (2)	C15—C14—H14	120.6
C2—C1—N1	122.9 (2)	C10—C15—C14	122.0 (2)
C6—C1—N1	116.7 (2)	C10—C15—H15	119.0
C3—C2—C1	120.0 (2)	C14—C15—H15	119.0
C3—C2—H2A	120.0	O2—C16—H16A	109.5
C1—C2—H2A	120.0	O2—C16—H16B	109.5
C4—C3—C2	120.5 (2)	H16A—C16—H16B	109.5
C4—C3—H3	119.8	O2—C16—H16C	109.5
C2—C3—H3	119.8	H16A—C16—H16C	109.5
C3—C4—C5	119.4 (2)	H16B—C16—H16C	109.5
C3—C4—H4	120.3	C9—C17—C18	111.45 (18)
C5—C4—H4	120.3	C9—C17—H17A	109.3
C4—C5—C6	122.0 (2)	C18—C17—H17A	109.3
C4—C5—H5	119.0	C9—C17—H17B	109.3
C6—C5—H5	119.0	C18—C17—H17B	109.3
C5—C6—C1	117.8 (2)	H17A—C17—H17B	108.0
C5—C6—C7	121.4 (2)	N4—C18—C17	111.24 (18)
C1—C6—C7	120.8 (2)	N4—C18—H18A	109.4
C6—C7—H7A	109.5	C17—C18—H18A	109.4
C6—C7—H7B	109.5	N4—C18—H18B	109.4
H7A—C7—H7B	109.5	C17—C18—H18B	109.4
C6—C7—H7C	109.5	H18A—C18—H18B	108.0
H7A—C7—H7C	109.5	C20—C19—N4	106.7 (2)
H7B—C7—H7C	109.5	C20—C19—H19	126.6
O1—C8—N1	125.5 (2)	N4—C19—H19	126.6
O1—C8—N2	121.4 (2)	C19—C20—N5	110.2 (2)
N1—C8—N2	113.09 (19)	C19—C20—H20	124.9
N3—C9—C10	115.8 (2)	N5—C20—H20	124.9
N3—C9—C17	124.0 (2)	N5—C21—N4	113.2 (2)
C10—C9—C17	120.22 (19)	N5—C21—H21	123.4
C15—C10—C11	118.1 (2)	N4—C21—H21	123.4
C15—C10—C9	121.5 (2)		
			
C8—N2—N3—C9	176.0 (2)	C15—C10—C11—C12	−0.7 (3)
C8—N1—C1—C2	−7.1 (4)	C9—C10—C11—C12	179.8 (2)
C8—N1—C1—C6	174.0 (2)	C10—C11—C12—C13	−0.5 (3)
C6—C1—C2—C3	0.8 (4)	C16—O2—C13—C14	6.7 (3)
N1—C1—C2—C3	−178.1 (2)	C16—O2—C13—C12	−173.1 (2)
C1—C2—C3—C4	−0.5 (4)	C11—C12—C13—O2	−179.7 (2)
C2—C3—C4—C5	0.2 (4)	C11—C12—C13—C14	0.5 (3)
C3—C4—C5—C6	−0.1 (4)	O2—C13—C14—C15	−179.2 (2)
C4—C5—C6—C1	0.4 (4)	C12—C13—C14—C15	0.6 (3)
C4—C5—C6—C7	179.0 (2)	C11—C10—C15—C14	1.8 (3)
C2—C1—C6—C5	−0.7 (3)	C9—C10—C15—C14	−178.7 (2)
N1—C1—C6—C5	178.2 (2)	C13—C14—C15—C10	−1.8 (4)
C2—C1—C6—C7	−179.3 (2)	N3—C9—C17—C18	−81.8 (3)
N1—C1—C6—C7	−0.3 (3)	C10—C9—C17—C18	96.8 (2)
C1—N1—C8—O1	−11.9 (4)	C21—N4—C18—C17	−130.0 (2)
C1—N1—C8—N2	168.7 (2)	C19—N4—C18—C17	50.5 (3)
N3—N2—C8—O1	−171.8 (2)	C9—C17—C18—N4	−179.38 (18)
N3—N2—C8—N1	7.6 (3)	C21—N4—C19—C20	0.1 (3)
N2—N3—C9—C10	−179.8 (2)	C18—N4—C19—C20	179.7 (2)
N2—N3—C9—C17	−1.1 (4)	N4—C19—C20—N5	0.3 (3)
N3—C9—C10—C15	154.6 (2)	C21—N5—C20—C19	−0.6 (3)
C17—C9—C10—C15	−24.1 (3)	C20—N5—C21—N4	0.7 (3)
N3—C9—C10—C11	−25.8 (3)	C19—N4—C21—N5	−0.5 (3)
C17—C9—C10—C11	155.4 (2)	C18—N4—C21—N5	179.9 (2)

### Hydrogen-bond geometry (Å, º)

Cg2 and Cg3 are the centroids of the C1–C6 and C10–C15 benzene rings, respectively.

**Table:**

*D*—H···*A*	*D*—H	H···*A*	*D*···*A*	*D*—H···*A*
N1—H1*n*···N3	0.87 (3)	2.04 (2)	2.568 (3)	118 (2)
N2—H2*n*···N5^i^	0.87 (3)	2.17 (3)	3.029 (3)	171 (2)
C16—H16*B*···O1^ii^	0.98	2.44	3.398 (3)	165
C20—H20···O1^i^	0.95	2.51	3.226 (3)	133
C17—H17*A*···*Cg*2^iii^	0.99	2.80	3.391 (3)	119
C18—H18*B*···*Cg*3^iv^	0.99	2.78	3.569 (2)	137

Symmetry codes: (i) −*x*+1, −*y*+1, −*z*+1; (ii) −*x*+2, *y*+1/2, −*z*+3/2; (iii) −*x*+2, −*y*+1, −*z*+2; (iv) −*x*+2, −*y*+1, −*z*+1.
